# SHP-2-induced M2 polarization of tumor associated macrophages *via* IL-4 regulate colorectal cancer progression

**DOI:** 10.3389/fonc.2023.1027575

**Published:** 2023-01-26

**Authors:** Zhihan Li, Jinchuan Xi, Baokun Li, Youqiang Liu, Guiying Wang, Bin Yu, Hongqing Ma, Zhilin Li, Zhenya Zhang

**Affiliations:** ^1^ Department of General Surgery, The Fourth Hospital of Hebei Medical University, Shijiazhuang, China; ^2^ Department of Gastrointestinal Surgery, The Third Hospital of Hebei Medical University, Shijiazhuang, China; ^3^ School of Basic Medicine, Hebei Medical University, Shijiazhuang, China

**Keywords:** Src homology region 2 domain-containing protein tyrosine phosphatase-2, tumor-associated macrophages, colorectal cancer, migration, invasion

## Abstract

**Objective:**

To explore the effect and molecular mechanism of Src homology region 2 domain-containing protein tyrosine phosphatase-2 (SHP-2) in tumor-associated macrophages (TAMs) repressing the migration and invasion of colorectal cancer (CRC) cells.

**Methods:**

The relevant data sets of human colon specimens were obtained from GEO database, and then the performed correlation analysis, principal component analysis and differentially expressed gene (DEGs) analysis on the samples were conducted. GO and KEGG enrichment analysis were performed on the common DEGs, and then functional interaction prediction was performed to verify the gene regulatory circuit of SHP-2. Furthermore, western blot was used to detect the effect of low expression of SHP-2 on related proteins, including the markers of promoting M2 polarization and exosome secretion, and keys proteins of the PI3K pathway. The relationship between SHP-2 and PI3K pathway was further verified by adding PI3K inhibitor LY294002. Finally, the effect of SHP-2 on the function of colon cancer cells was confirmed by wound healing assay and Transwell assay.

**Results:**

Through bioinformatics analysis, SHP-2 was screened as a possible key gene affecting CRC. The low expression of SHP-2 promoted the protein levels of Arginase-1 and IL-10 in IL-4 induced M2 macrophages, while inhibited the protein levels of IL-1β and TNF-α. Meanwhile, low expression of SHP-2 was found to similarly promote the expression of p-PI3K, p-AKT, and the release of exosomes. Interestingly, the promotion was suppressed after the addition of the PI3K inhibitor LY294002. In terms of cellular behavior, wound healing and transwell data showed that low expression of SHP-2 enhanced the migration and invasion abilities of CRC cells.

**Conclusion:**

The low expression of SHP-2 induced by PHPS1 may regulate M2 polarization of TAMs and release of exosomes through PI3K/AKT pathway, thereby enhancing the migration and invasion ability of CRC cells.

## Introduction

Colorectal cancer (CRC) is one of the digestive tract malignant tumors posing serious threats to human life safety, and its incidence ranks fourth in the global malignant tumors (1). The primary cause for the death of CRC patients lies in the sustaining and unlimited proliferation, infiltration and metastasis of CRC cells (2). Despite the certain progress of molecular targeted drug-chemotherapy combined therapeutic scheme in recent years, CRC patients already subjected to metastasis still suffer from an unsatisfactory prognosis (3, 4). Hence, it is of great significance to uncover the molecular mechanisms of CRC metastasis and determine new molecular targets in the development of novel and more effective treatments for this deadly malignancy.

Src homology region 2 domain-containing protein tyrosine phosphatase-2 (SHP-2) is encoded by PTPN11 gene. Belonging to tyrosine phosphatase family, SHP-2 is a key regulatory factor in signal transduction, which is affected by cytokines and extracellular stimuli and widely expressed in all kinds of cells and tissues (5). SHP-2 mutation could cause Noonan syndrome (6), adolescent granular monocyte leukemia (7) and other human diseases. Additionally, SHP-2 also plays an important role in various human malignancies. SHP-2 is generally considered as an oncogene, because it can enhance the malignancy of many types of cancer. However, whether SHP-2 acts as a tumor-promoting or tumor-suppressing molecule is determined by the cell types (8). For example, SHP-2 functions as a tumor suppressor in normal hepatocytes, but its expression in liver cancer cells can enhance the RAS-ERK and PI3K-AKT-mTOR signaling pathways to promote tumorigenesis (9). Yang’s group has revealed that in these chondrocyte progenitors, SHP-2 deficiency inhibits ERK activation but promotes Hedgehog signaling, resulting in over-proliferation of chondrocytes (10). Coincidentally, another research group has also reported that targeted disruption of SHP-2 in chondrocytes triggers metachondromatosis with multiple cartilaginous protrusions (11). In colon cancer, SHP-2 expression is significantly reduced in colon tumor tissues when compared with normal colon tissues, and SHP2 expression is negatively correlated with tumor differentiation and progression (12). However, SHP-2 in CD4^+^ T cells acts a positive regulator of the occurrence of colitis-associated colorectal cancer (13). Given the prominent role of SHP-2 in various tumors, the exploration of the relationship between SHP-2 and CRC has been highlighted as a potential new direction to conquer CRC.

Existing data suggest that the tumor development is not only a result of changes in tumor cytogenetics and epigenetics, but also an interaction with tumor microenvironments (TMEs) (14). TMEs are mainly composed of cancer cells, cancer-associated fibroblasts, immune cells and the non-cellular components, among which immune cells like T lymphocytes and macrophage have received a lot of attention due to their outstanding positive effects (15). In T lymphocytes, various immunosuppressive receptors, such as PD-1, BTLA, CTLA-4 and TIGIT, could recruit SHP-2 through their specific phosphotyrosine motifs, thereby regulating the activation of T lymphocytes (8, 16). For example, SHP-2 binds to the ITSM of the immune checkpoint protein PD-1 through its two tandem Src homology domains, activating SHP-2-mediated immunosuppression (17). In tumor-associated macrophages (TAMs), SHP-2 binds to the signaling protein complex of growth factor receptor-bound protein 2/GRB2 associated binding protein-2 (GRB2/GAB2), which is induced by colony stimulating factor receptor under the stimulation of CSF-1, and promotes macrophage proliferation and M2-type polarization. It is widely believed that cells from the TMEs contribute to tumor metastasis (18, 19). Accumulating evidence suggests that exosomes play key roles in remodeling the TME and tumor metastasis by transferring signal peptides, noncoding RNAs, or DNA to neighboring cells or tissues (20). All these studies indicate SHP-2 as a potential target to regulate TMEs for cancer immunotherapy.

In the light of the roles of SHP-2 in promoting multifarious malignant behaviors of tumor cells, the development of molecular inhibitors has attracted extensive attention (17, 21). Traditional inhibitors bind to the catalytic PTP pocket, preventing the substrates of tyrosine phosphorylation from entering the catalytic sites, thus inhibiting the phosphatase activity of SHP-2. Phenylhydrazonopyrazolone sulfonate 1 (PHPS) 1, as a potential phosphotyrosine inhibitor, displayed strong inhibition on SHP-2. It delayed the development of DMBA-induced tumors in the rat mammary gland and also blocked tumor formation in MMTV-pyvt transgenic mice (22). Facing the challenge of limited selectivity and cell permeability, a possible solution is not dependent on allosteric inhibitors binding to phosphotyrosine binding sites (23). SHP099 is the first allosteric inhibitor that acts as “molecule glue” which selectively blocks SHP-2 activity by locking it in an auto-inhibited conformation. Compelling evidence suggested that SHP099 retards tumor growth through triggering CD8^+^ T cell-mediated anti-tumor immunity and synergizes with PD-1 blockade in murine colon xenograft model (24).

In view of the crucial roles of TEMs in dictating CRC metastasis and for another allosteric inhibitor SHP099 is known to inhibit cancer cell growth both *in vitro* and *in vivo*, we speculated that the crosstalk between TAMs and tumor cells could affect the tumor metastasis. Hence, this study aims to explore the molecular mechanism of SHP-2 in TAMs in repressing the migration and invasion of CRC cells, hoping to facilitate the screening, diagnosis and treatment of CRC.

## Materials and methods

### Bioinformatics analysis

CRC-related datasets were searched from GENE EXPRESSION OMNIBUS (GEO) database (https://www.ncbi.nlm.nih.gov/gds/). Then, CRC-related mRNA gene expression datasets GSE122183 and GSE128435 were found and downloaded. Next, quantile normalization was performed for RNA-sequencing (RNA-seq) data of GSE122183 *via* Limma software package of R language, followed by sample correlation analysis, principal component analysis (PCA) and differentially expressed gene (DEGs) analysis (|logFC|>1, *p*<0.05). Afterwards, a volcano plot of visually grouped DEGs in GSE122183 was established using ggplot2 package of R software, and a cluster analysis heatmap of DEGs was drawn through pheatmap package of R software. Similarly, quantile normalization was done for RNA-seq data of GSE128435 using Limma software package of R language, followed by sample correlation analysis, PCA and DEG analysis (|logFC|>1, *p*-value<0.05). Subsequently, a volcano plot of visually grouped DEGs in GSE128435 was constructed using ggplot2 package of R software and a cluster analysis heatmap of DEGs was drawn through pheatmap package of R software.

### Functional enrichment analysis

The intersection between GSE122183 and GSE128435 was extracted *via* RobustRankAggreg package of R language to acquire their common DEGs. Then, Gene Ontology (GO) enrichment analysis and Kyoto Encyclopedia of Genes and Genomes (KEGG) enrichment analysis were conducted on these common DEGs. Moreover, the corresponding DEGs at the biological process, cellular component and molecular function levels were analyzed through DAVID online database tool (https://david.ncifcrf.gov) to integrate GO terms and create the biological process network of DEGs. Next, a GO pathway diagram and a KEGG pathway enrichment analysis diagram of DEGs were drawn in R language using GOplot and ggplot2 packages.

### Protein-protein interaction network analysis of mRNA DEGs and target gene screening

DEGs were input into STRING online tool to screen out interacting proteins with a combined score >0.9. According to |LogFC|>1 and the Padj value<0.05, the sub-network of hub genes were constructed. Hub genes were calculated using the degree algorithm, and sorted according to the degree score, represented by color (red) and size, that is, the higher the degree score, the darker the color and the larger the corresponding icon.

### Cell culture and treatment

Human THP-1 macrophages were stationarily cultured with RPMI-1640 medium (brand: Gibco, item No.: C11875500BT) containing 10% FBS (Gibco, 10099-141), 10 U/mL penicillin and 10 μg/mL streptomycin in a 5% CO_2_ incubator at 37°C. In the culture process, the cell count was kept at 1×10^7^/culture flask. Next, the sub-cultured THP-1 cells were diluted into 1×10^6^/mL, inoculated in a 35 mm culture dish and cultured in low-serum RPMI-1640 medium containing 100 ng/mL phorbol-12-myristate-13-acetate (PMA) (MCE, HY-18739) and 0.3% BSA to induce their differentiation into M0 macrophages. Furthermore, 10 μm of SHP-2 inhibitors PHPS1 (brand: MCE, HY-112368) and/or 15 ng/mL IL-4 (MCE, HY-P70445) and/or 30 μM of PI3K inhibitors LY294002 (MCE, HY-10108) was added into the medium and incubated for 24 hours. According to experimental requirements, the treated cells were divided into control group, PHPS1 group, IL-4 group, PHPS1+IL-4 group, IL-4+ LY294002 group and PHPS1+IL-4+LY294002 group.

CRC cell lines SW480 and HCT116 were purchased from Nanjing KeyGen BioTech Co., Ltd. After being intervened by PHPS1 and/or IL-4 for 24 hours, macrophages were centrifuged and co-cultured with CRC cell lines SW480 and HCT116. Then, the phenotypes of CRC cells were experimentally observed.

### Western blotting

The macrophages in each group were collected, from which the total proteins were extracted, and their concentrations were detected through BCA method. Then, the extracted proteins were mixed well with 4×loading buffer and boiled in a metal bath at 100°C for 10 min, followed by SDS-PAGE. Next, the proteins were transferred to a PVDF membrane and incubated at 4°C overnight against the primary antibodies: IL-6 (Abcam, ab233706, 1:1000), Arginase-1 (Abcam, ab133543, 1:1000), IL-1β (Abcam, ab254360, 1:1000), tumor necrosis factor-α (TNF-α, Abcam, ab215188, 1:1000), p-phosphatidylinositol 3-kinase (p-PI3K, Abcam, ab278545, 1:1000), p-protein kinase B (p-AKT, Abcam, ab38449, 1:800), cluster of differentiation 9 (CD9, Abcam, ab236630, 1:1000), CD81 (Abcam, ab109201, 1:5000), CD63 (Abcam, ab134045, 1:5000) and GAPDH (Abcam, ab8245, 1:5000). After rinsing, they were incubated in the secondary antibody working solution of horseradish peroxidase-labeled goat anti-rabbit IgG (Abcam, ab97080, 1:5000) at room temperature for 2 hours. Subsequently, exposure and development were performed with enhanced chemiluminescent (ECL) solution, and the corresponding gray value was measured *via* Image J software.

### Wound healing assay

Macrophages of each group were added in the upper chamber of the co-culture system (pore size: 0.4 μm, brand: Corning, item No.: 3412). In the lower chamber, 2×10^5^ SW480 and 2×10^5^ HCT116 cells were respectively inoculated into a 24-well plate and stayed overnight in a thermostatic incubator containing 5% CO_2_ at 37°C. When cells overgrew at 2 days, the cells in the lower chamber were scratched in the middle through a 200 μL pipette tip to make a “wound”. In addition, the medium in upper and lower chambers was replaced by medium containing 1% FBS. At 0, 24 and 48 hours after scratching, the wound conditions were recorded by taking pictures under an inverted microscope, and the scratch width was measured using the measuring tool of Image J (Fiji, V1.49, open source). In detail, images were normalized in terms of white balance and transformed in Binomial and image closure was calculated on the raw light intensity per image. Raw light intensity was different for every image stack. To normalize the values between images stacks, the light intensity was calculated in percentage from 0% to maximum 100%. The reduction of the gap due to the migrating cells was scored in every image stack and recorded with a specific software tool (Time Series Analyzer, V3.0). Speed of closure was expressed in µm^2^/min, measured on the basis of the percentage of the cell closure during time, considering that the initial measured area of the gap was of 1 mm^2^.

### Transwell assay

For migration, before the cell suspension was prepared, serum starvation of cells was performed for 12 hours to eliminate the influence of serum. Next, 100 μL of serum-free SW480 and HCT116 cell suspensions containing 1×10^5^ cells were inoculated in the upper Transwell (pore size: 8 μm, brand: Corning, item No.: 3422) chamber. In the lower chamber, 700 μL of serum-free cell suspension containing 2×10^5^ macrophages in each group was added and cultured for 48 hours. The cells in Transwell chambers were fixed using 4% paraformaldehyde, followed by 4% crystal violet staining. Then, the cells adhering to the upper chamber surface of the bottom membrane were wiped away with a cotton swab, while those on the lower chamber surface were reserved. For invasion assay, the upper Transwell chamber was coated with Matrigel, while the other steps remained the same as those of migration assay. Cells were quantified in five randomly selected fields for each membrane, and the average cell count for three individual membranes was defined as the migration or invasion index.

### Statistical processing

Rv3.6.1 software packages DEseq2 and ggpubr were used for bioinformatics analysis. Wald test was adopted for DEG analysis and rank-sum test for comparison of cytokines between two groups. Other indexes were statistically analyzed *via* SPSS23.0. Measurement data were expressed as mean ± standard deviation, and one-way analysis of variance was applied to intergroup comparisons and Least Significant Difference (LSD) test to pairwise comparisons. Survival was analyzed *via* Kaplan-Meier (KM) survival curves. *P*<0.05 indicated that the difference was statistically significant.

## Results

### Screening of DEGs

**Figure 1:**
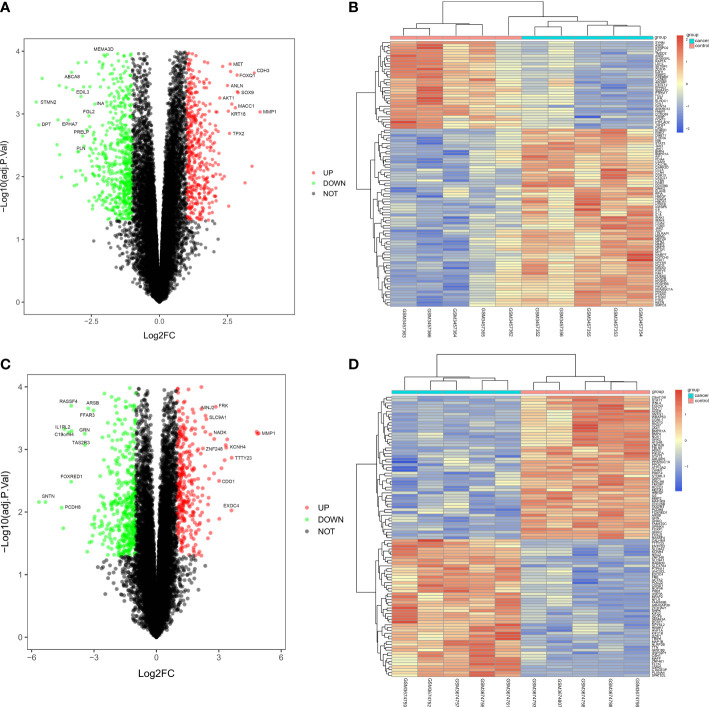
Screening of DEGs. **(A, B)** Volcano plot and heatmap of DEGs in GSE122183; **(C, D)** Volcano plot and heatmap of DEGs in GSE128435. DEGs, differentially expressed genes.

The CRC-related dataset GSE122183 was downloaded from GEO database, followed by quantile normalization of data. As per criteria of *P*<0.05 and |logFC|>1, a total of 562 DEGs in the mRNAs of CRC were screened from GSE122183, including 276 up-regulated ones and 286 down-regulated ones. Next, a volcano plot (**Figure 1A**) of visually grouped DEGs showing the top 10 DEGs with significant up-regulation and down-regulation in GSE122183 was established using ggplot2 package of R software, and a cluster analysis heatmap (**Figure 1B**) of the first 100 DEGs was drawn using pheatmap package of R software. Similarly, the dataset GSE128435 was downloaded followed by quantile normalization of data. According to criteria of *P*<0.05 and |logFC|<1, a total of 767 DEGs in the mRNAs of CRC were screened from GSE128435, including 333 up-regulated DEGs and 434 down-regulated DEGs. Next, a volcano plot (**Figure 1C**) of visually grouped DEGs, displaying the top 10 DEGs with significant up-regulation and down-regulation in GSE128435 was established using ggplot2 package of R software, and a cluster analysis heatmap (**Figure 1D**) of the first 100 DEGs was drawn using pheatmap package of R software.

### Bioinformatics analysis

**Figure 2:**
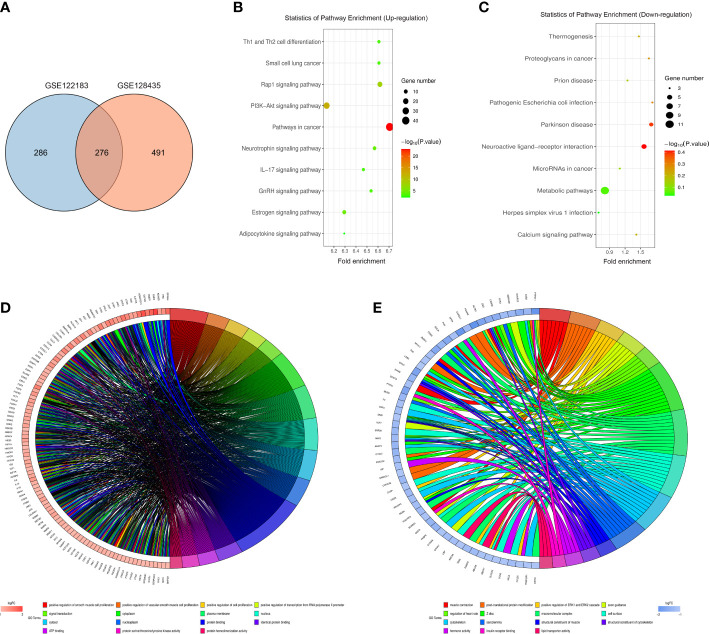
Bioinformatics analysis. **(A)** Common DEGs obtained from GSE122183 and GSE128435 were represented *via* Venn diagram; **(B, C)** Up-regulation and down-regulation KEGG pathway diagrams of DEGs were drawn *via* KEGG pathway analysis; **(D, E)** Biological process network of up-regulated DEGs and down-regulated DEGs. GO, Gene Ontology; KEGG, Kyoto Encyclopedia of Genes and Genomes; DEGs, differentially expressed genes.

The intersection between GSE122183 and GSE128435 was extracted *via* RobustRankAggreg package of R language. In this way, common DEGs were obtained, including 115 up-regulated ones and 161 down-regulated ones (**Figure 2A**). Then, GO enrichment analysis and KEGG enrichment analysis of these common DEGs was carried out. Next, the DEGs at the biological process level were analyzed through DAVID online database tool to integrate GO terms and create the biological process network of DEGs. As depicted in **Figure 2D** and **Table 1**, the up-regulated DEG participated in the biological process like positive regulation of smooth muscle cell proliferation, positive regulation of vascular smooth muscle cell proliferation and positive regulation of cell proliferation. Biological processes such as muscle contraction, post-translational protein modification and positive regulation of ERK1 and ERK2 cascade were involved in the down-regulated DEG regulation (**Figure 2E** and **Table 2**). Subsequently, KEGG pathway analysis of DEGs was performed and the KEGG pathway diagrams of up-regulation and down-regulation were drawn. It was found that pathways in cancer, PI3K/AKT signaling pathway and mammalian target of rapamycin (mTOR) signaling pathway were the enriched pathways (**Figures 2B, C**).

**Table 1:** Up-regulated differentially expressed genes in biological process.

GO Terms	Up-regulated Genes
Positive regulation of smooth muscle cell proliferation	CX3CL1, IGF1R, SMPD3, MYC, NAMPT, CCN4, AKT1, SKP2, PDGFRB, EDN1, NPY5R, ITGA2, MMP2, IL13, SERPINF2, IL18, CYBA, IRAK4, EREG, FOXP1, PIK3CA, CDH13, HBEGF, HDAC4, NOTCH3, CALCRL, PTAFR, PDGFB, ALOX12, HMGCR, PTGS2, AIF1, IRAK1, PDGFD, CCL5, S1PR1, HMOX1, PPARGC1A, ELANE, HES5, STAT1, IGF1, MTOR, BMP4, IL6, NR4A3, RPS6KB1, ID2, MNAT1, MYD88, NOX1, FGFR2
Signal transduction	GSK3B, SHC1, RETN, FGF2, HIF1A, GNAI2, IGF1R, GRK2, FGF9, NAMPT, CCN4, AKT1, JAK2, PDGFRB, IGFBP5, STAT1, PDE4D, IL13, STAT3, IGF1, IRAK4, OLFM4, EREG, NR4A3, RPS6KB1, MYD88, NOX1, HBEGF, DDR2
Cytosol	HDAC4, GSK3B, NOTCH3, CAMK2D, HPGD, SHC1, ALOX12, AIF1, HIF1A, GNAI2, PAK1, GRK2, IRAK1, ORC1, NAMPT, MFN2, CCN4, AKT1, HMOX1, JAK2, LDLRAP1, SKP2, JAK3, ELANE, JAK1, JUN, NQO2, STAT1, PDE4D, STAT3, IL18, FRS2, PRKCA, PTK6, NMI, IRAK4, OLFM4, MTOR, PTK2, AGT, FER, PIK3CA, RPS6KB1, ID2, MDM2, MYD88, FRK
ATP binding	GSK3B, CAMK2D, FLT1, IGF1R, PAK1, GRK2, IRAK1, ORC1, AKT1, JAK2, JAK3, JAK1, PDGFRB, PRKCA, PTK6, IRAK4, MTOR, PTK2, ERN1, FER, PIK3CA, DDX39B, RPS6KB1, FRK, FGFR2, BMPR1A, DDR2
Positive regulation of vascular smooth muscle cell proliferation	DNMT1, HPGD, GNAI3, PDGFB, FGF2, GNAI2, PAK1, P2RY6, FGF9, ADAMTS1, MFN2, JAK2, LDLRAP1, MEF2D, JUN, NQO2, EDN1, IGFBP5, MMP2, FOXJ2, HTR1B, FRS2, IGF1, MMP9, AGT, ERN1, NR4A3, DDX39B, MDM2, BMPR1A
Cytoplasm	GSK3B, FGF2, IGF1R, SMPD3, FGF9, DMBT1, NAMPT, AKT1, JAK2, JAK1, PDGFRB, EDN1, NPY5R, IL13, IL18, FRS2, PRKCA, IRAK4, FOXP1, ERN1, PIK3CA, DDX39B, CDH13, HDAC4, CAMK2D, CALCRL, HPGD, SHC1, PDGFB, GNAI3, ALOX12, PTGS2, HIF1A, AIF1, GNAI2, PAK1, GRK2, IRAK1, S1PR1, IFNL4, PPARGC1A, MEF2D, ELANE, EGR1, STAT1, STAT3, HTR1B, PTK6, NMI, MTOR, PTK2, FER, RPS6KB1, ID2, GNAQ, MDM2, MYD88, FGFR2
Nucleoplasm	HDAC4, GSK3B, NOTCH3, CAMK2D, DNMT1, KDM5D, HPGD, ITGB3, CTR9, HIF1A, GNAI2, PAK1, IRAK1, ORC1, MYC, S1PR1, AKT1, HMOX1, JAK2, SKP2, PPARGC1A, MEF2D, HES5, EGR1, JUN, NQO2, STAT1, STAT3, FOXJ2, PRKCA, PTK6, NMI, OLFM4, MTOR, FOXP1, NR4A3, DDX39B, RPS6KB1, ID2, MDM2, MNAT1, FRK
Protein serine/threonine/tyrosine kinase activity	PDGFRB, GSK3B, CAMK2D, FLT1, PRKCA, PTK6, IRAK4, MTOR, PTK2, IGF1R, ERN1, FER, PAK1, IRAK1, PIK3CA, RPS6KB1, AKT1, JAK2, JAK3, FRK, FGFR2, JAK1, DDR2
Positive regulation of cell proliferation	HDAC4, FLT1, SHC1, PDGFB, FGF2, AIF1, CX3CL1, GNAI2, IGF1R, PAK1, FGF9, MYC, PDGFD, NAMPT, AKT1, HES5, PDGFRB, EDN1, IL31RA, IGF1, PTK2, EREG, BMP4, FER, IL6, MDM2, FGFR2, NOX1, HBEGF
Plasma membrane	GSK3B, FLT1, ITGB3, CX3CL1, IGF1R, SMPD3, NAMPT, C3AR1, AKT1, JAK2, LDLRAP1, JAK3, PDGFRB, NPY5R, PDE4D, ITGA2, MMP2, FRS2, PRKCA, CYBA, IRAK4, OLFM4, PIK3CA, CDH13, HBEGF, DDR2, NOTCH3, CALCRL, SHC1, PTAFR, GNAI3, ALOX12, AGER, GNAI2, P2RY6, PAK1, GRK2, IRAK1, S1PR1, JUN, STAT3, IL31RA, HTR1B, PTK6, PTK2, FER, GNAQ, MDM2, MYD88, NOX1, FGFR2, BMPR1A
Protein binding	GSK3B, PDGFB, AKT1
Protein homodimerization activity	NQO2, CAMK2D, STAT1, SERPINF2, STAT3, PDGFB, PTGS2, ERN1, NR4A3, IRAK1, CCL5, AKT1, CDH13, HMOX1, MEF2D, FGFR2, BMPR1A
Positive regulation of transcription from RNA polymerase II promoter	HDAC4, NOTCH3, FGF2, HIF1A, CX3CL1, MYC, NAMPT, S1PR1, AKT1, JAK2, PPARGC1A, MEF2D, HES5, EGR1, JUN, EDN1, STAT1, SERPINF2, STAT3, FOXJ2, IL18, IGF1, BMP4, IL6, NR4A3, MDM2, CDH13, FGFR2, BMPR1A
Nucleus	GSK3B, KDM5D, ITGB3, FGF2, IGF1R, EME2, MYC, NAMPT, AKT1, JAK2, SKP2, JAK1, PDGFRB, PDE4D, ITGA2, MMP2, PRKCA, CYBA, IRAK4, FOXP1, DDX39B, HDAC4, CAMK2D, DNMT1, RETN, HIF1A, AIF1, ORC1, IRAK1, HMOX1, PPARGC1A, MEF2D, HES5, EGR1, JUN, STAT1, STAT3, FOXJ2, PTK6, NMI, MTOR, PTK2, FER, NR4A3, RPS6KB1, ID2, MDM2, MYD88, FRK, FGFR2
Identical protein binding	HDAC4, NOTCH3, CAMK2D, HPGD, ITGB3, PDGFB, HMGCR, FGF2, AGER, IGF1R, SMPD3, PAK1, IRAK1, CCL5, NAMPT, AKT1, HMOX1, JAK2, SKP2, JUN, STAT1, STAT3, FOXJ2, PTK6, NMI, MMP9, MTOR, FOXP1, ERN1, DDX39B, RPS6KB1, MDM2, MYD88, FGFR2

**Table 2:** Down-regulated differentially expressed genes in biological process.

GO Terms	Down-regulated Genes
Muscle contraction	ACTA2, DES, SGCA, SCN7A, MYH11, SORBS1, MYH7
Regulation of heart rate	CASQ2, DMD, ANK2, BVES, MYH7
Cytoskeleton	CNN1, SGCA, ARL6IP5, LDB3, ITGB1BP1, DMD, ANK2, AKAP4, ASB2, S100A8
Hormone activity	CCL25, UCN3, NPPA, VIP
Post-transiational protein modification	PSMA3, TNC, COPS7B, SPARCL1, FBXO32, ASB2, CHRDL1
Z disc	SYNPO2, NEXN, LDB3, ANK2, AKAP4, FBXO32, RYR3, DES, CASQ2, PGM5, DMD, ASB2, MYH7
Sarcoemma	SYNM, DES, SGCA, PGM5, DMD, ANK2, BVES, RYR3
Insulin receptor binding	PTPN1, PTPN11, SORBS1
Positive regulation of ERK1 and ERK2 cascade	CCL25, ACTA2, CASR, RAMP3, PTPN11, ABCA7
Macromolecular complex	ACTA2, PTPN1, PGLYRP3, ZNRF2, PLN, RFTN1, NPPA, FKBP8, PTPN11, DMD, TBX5, C1QL2
Structural constituent of muscle	SYNM, NEXN, MYH11, DMD
Lipid transporter activity	ABCA6, ABCA7, ABCA8
Axon guidance	KLF7, NGFR, EPHA7, SEMA3D, NEXN, PTPN11
Cell surface	NGFR, CASR, CLMP, RAMP3, ERP29, ABCA7, DMD, LBP, F3, MICA, SLC6A3
Structural constituent of cytoskeleton	SYNM, DES, DMD, ANK2

### PPI network analysis of DEGs and target gene screening

**Figure 3:**
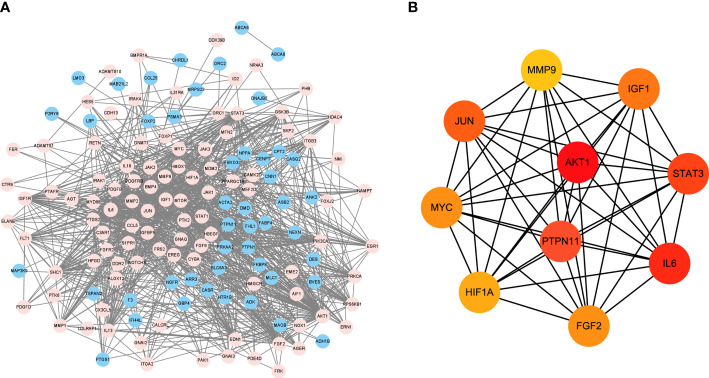
PPI network analysis of DEGs and target gene screening. **(A)** DEGs were imported into STRING database to obtain PPI network. Blue represents the down-regulation, and pink represents the up-regulation; **(B)** The sub-network of hub genes were constructed, and hub genes were calculated using the degree algorithm. DEGs, differentially expressed genes; PPI, protein-protein interaction.

DEGs were imported into STRING database to obtain PPI network (**Figure 3A**). Blue represents the down-regulation, and pink represents the up-regulation. Next, according to |LogFC|>1 and the Padj value<0.05, the sub-network of hub genes were constructed (10 genes). Hub genes were calculated using the degree algorithm, and sorted according to the degree score, represented by color (red) and size, that is, the higher the degree score, the darker the color and the larger the corresponding icon. PTPN11, also known as SHP-2, was among these nodes (**Figure 3B**).

### Survival analysis

**Figure 4:**
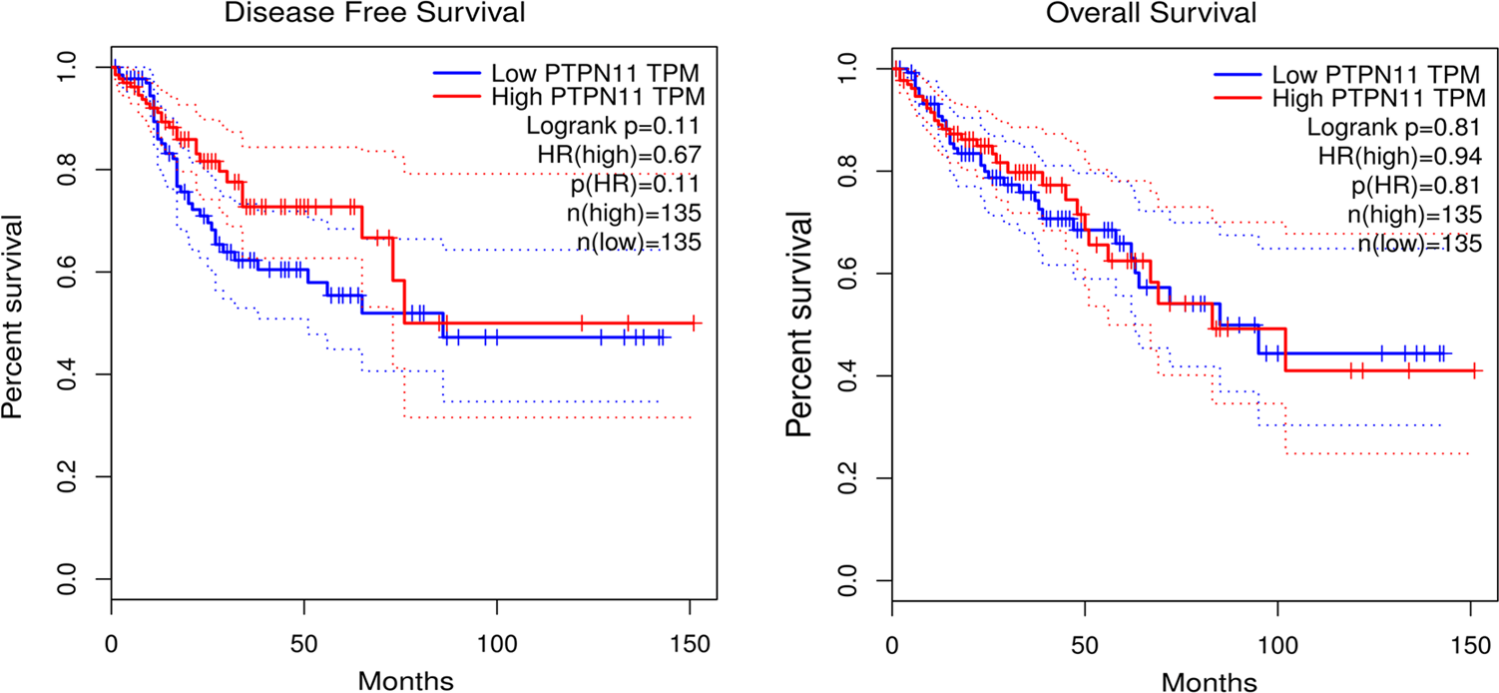
Survival analysis. Kaplan-Meier (KM) survival curves were used to represent disease free survival and overall survival of CRC patients. CRC, colorectal cancer.

As shown in **Figure 4**, both low and high expression SHP-2 did not affect the disease free survival and overall survival of CRC patients.

### Low SHP-2 expression promoted the protein levels of Arginase-1 and IL-10 and inhibited those of IL-1β and TNF-α in M2 macrophages

**Figure 5:**
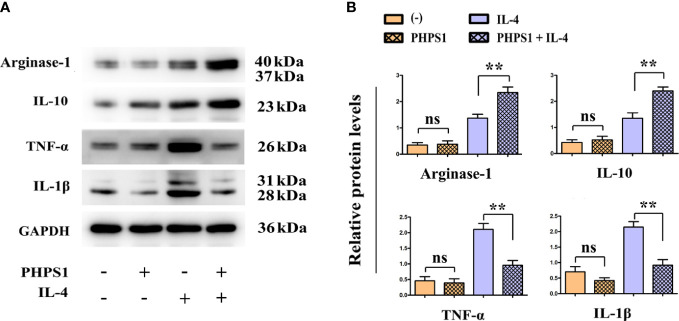
Low SHP-2 expression promoted the protein levels of Arginase-1 and IL-10 and inhibited those of IL-1β and TNF-α in M2 macrophages. **(A)** Western bolt was used to detect the protein levels of Arginase-1, IL-10, IL-1β and TNF-α; **(B)** Quantitative analysis of the protein expression of Arginase-1, IL-10, IL-1β and TNF-α. ns indicated no statistical difference; ^**^ *P*<0.01 indicated *vs.* IL-4 group. All experiments were independently repeated three times. SHP-2, Src homology region 2 domain-containing protein tyrosine phosphatase-2.

Compared with control group, we observed that low expression of SHP-2 promoted the protein expression of Arginase-1 and IL-10, while inhibited those of IL-1β and TNF-α, although there was no statistically significant difference. It was generally known that IL-4 can induce the differentiation of M2 macrophages. Consistently, the protein levels of Arginase-1 and IL-10 were observed to be significantly up-regulated in IL-4 group. Besides, it was found that the protein levels of Arginase-1 and IL-10 were evidently elevated, while those of IL-1β and TNF-α were obviously reduced in M2 macrophages in PHPS1+IL-4 group (**Figure 5**).

### Low SHP-2 expression facilitated the migration and invasion abilities of CRC cells co-cultured with M2 macrophages

**Figure 6:**
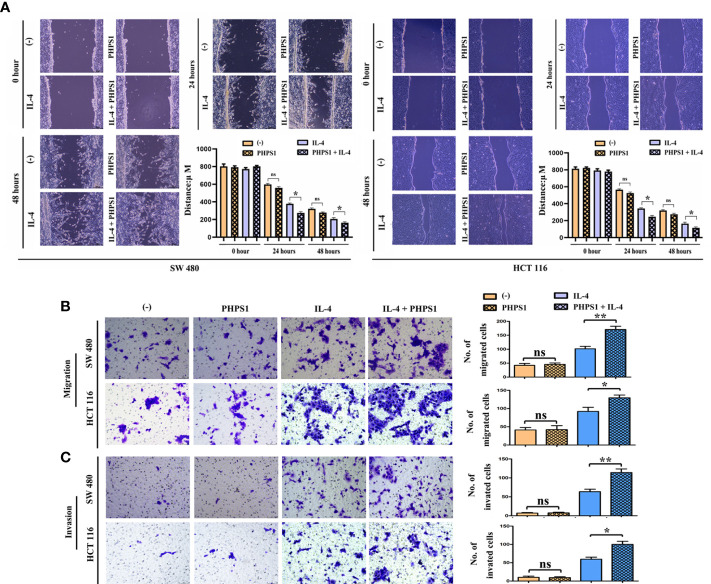
Low SHP-2 expression facilitated the migration and invasion abilities of CRC cells co-cultured with M2 macrophages. **(A)** Wound healing assay was used to evaluate the migration abilities of CRC cells; **(B)** Transwell assay was used to evaluate the migration abilities and **(C)** invasion abilities of CRC cells; ns indicated no statistical difference; ^*^P<0.05 or ^**^P<0.01 indicated vs. IL-4 group. All experiments were independently repeated three times. SHP-2, Src homology region 2 domain-containing protein tyrosine phosphatase-2; CRC, colorectal cancer.

The wound healing assay results showed that after 24 and 48 hours, the scratch width of CRC cells co-cultured with IL-4-treated macrophages was apparently smaller than that in control group, indicating that the migration rate of tumor cells to the center of scratch increased significantly. More interestingly, this migration rate increased even more after co-treatment with PHPS1 (**Figure 6A**). Furthermore, more migrated CRC cells in PHPS1+IL-4 group compared to IL-4 group further support this conclusion (**Figure 6B**). In Transwell assay, it was observed that the invaded SW480 cells and HCCT116 cells in the PHPS1+IL-4 group were significantly higher than those in the IL-4 group, indicating that the low SHP-2 expression induced by PHPS1 enhanced the invasive ability of CRC cells (**Figure 6C**).

### Low SHP-2 expression activated PI3K signaling pathway in M2 macrophages

**Figure 7:**
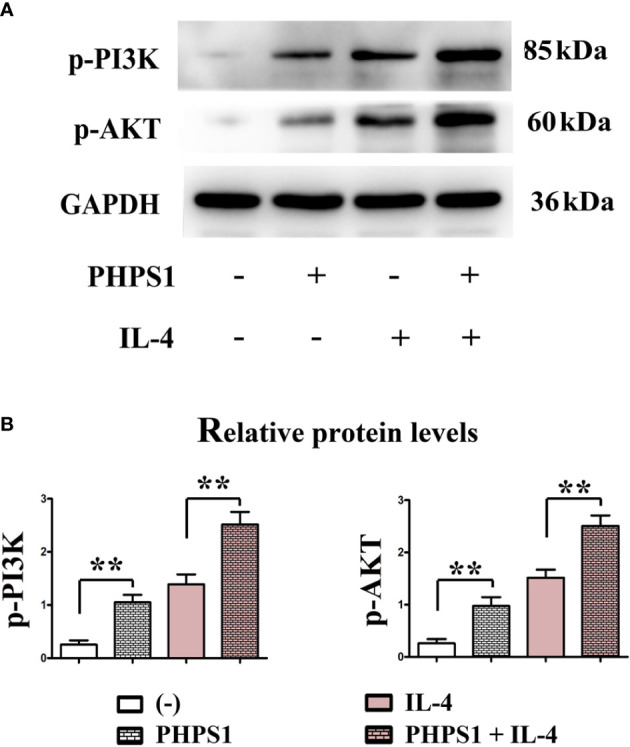
Low SHP-2 expression activated PI3K signaling pathway in M2 macrophages **(A, B)**. The protein expressions of p-PI3K and p-AKT in macrophages. All experiments were independently repeated three times. Src homology region 2 domain-containing protein tyrosine phosphatase-2. **indicated *P*<0.01.

**Figure 7** showed that compared with control group, the protein expressions of p-PI3K and p-AKT in macrophages rose evidently after intervention of PHPS1. Similarly, the protein expressions of p-PI3K and p-AKT in macrophages in PHPS1+IL-4 group were evidently increased in comparison with those in IL-4 group, manifesting that PHPS1 could promote the protein expressions of p-PI3K and p-AKT in macrophages. To further confirm the relationship between SHP-2 and PI3K/AKT signaling pathway in M2 macrophages, pathway inhibitor LY294002 was added. As expected, LY294002 significantly inhibited the activation of PI3K/AKT pathway induced by low expression of SHP-2, as indicated by a significant decrease in p-PI3K and p-AKT (**Figure 7**).

### Low SHP-2 expression may facilitated the release of exosomes in M2 macrophages through PI3K/AKT signaling pathway

**Figure 8:**
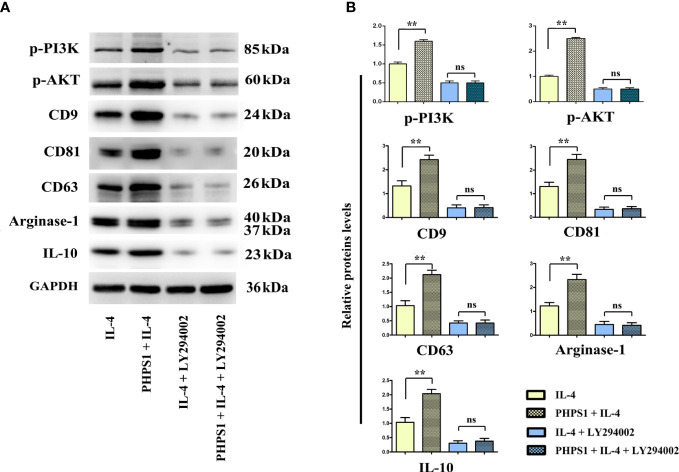
Low SHP-2 expression may facilitated the release of exosomes in M2 macrophages through PI3K/AKT signaling pathway. **(A)** Western bolt was used to detect the protein levels of p-PI3K, p-AKT, CD9, CD81, CD63, Arginase-1 and IL-10; **(B)** Quantitative analysis of the protein expression of p-PI3K, p-AKT, CD9, CD81, CD63, Arginase-1 and IL-10; ns indicated no statistical difference. All experiments were independently repeated three times. Src homology region 2 domain-containing protein tyrosine phosphatase-2; CRC, colorectal cancer. ns indicated no statistical difference; ** indicated *P*<0.01.

As depicted in **Figure 8**, compared with IL-4 group, the protein expressions of CD9, CD81, CD63, Arginase-1 and IL-10 of macrophages in PHPS1+IL-4 group grew obviously, manifesting that if expressed at a low level, SHP-2 can up-regulate the protein expressions of CD9, CD81, CD63, Arginase-1 and IL-1, further boosting the differentiation of M2 macrophages and facilitated the release of exosomes. After intervention of LY294002, it was discovered that in comparison to PHPS1+IL-4 group, the above proteins were evidently decreased in PHPS1+IL-4+LY294002 group, reflecting that low SHP-2 expression induced by PHPS1 may regulate the proliferation and differentiation of M2 macrophages and the release of exosomes by activating the PI3K/AKT pathway.

## Discussion

**Figure 9:**
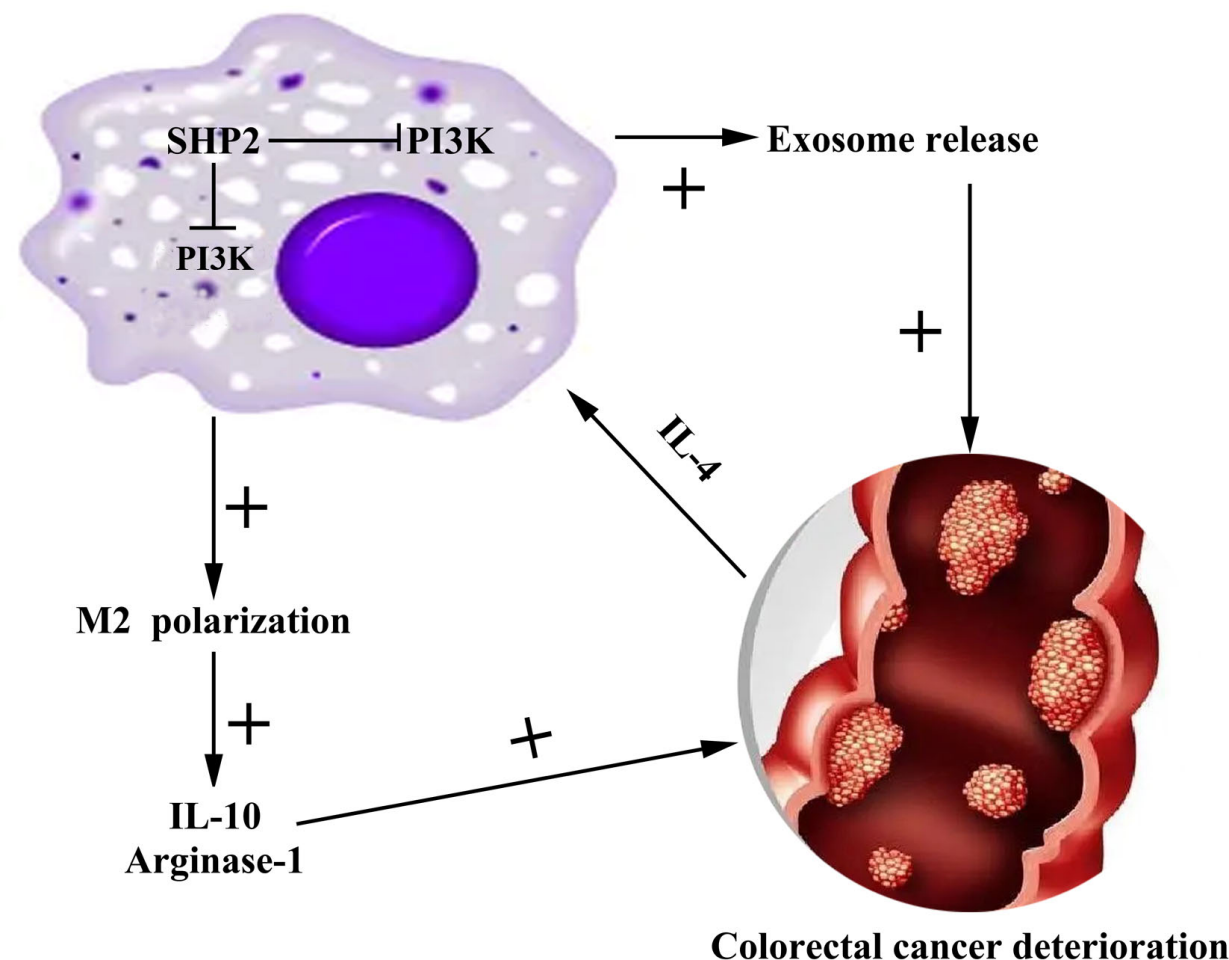
Mechanism diagram of SHP-2 regulating CRC through TAMs. Src homology region 2 domain-containing protein tyrosine phosphatase-2; CRC, colorectal cancer; TAMs tumor-associated macrophages.

As a digestive tract malignant tumor in the colon, CRC frequently occurs at the junction between rectum and sigmoid colon (25). CRC occurs in a staged and multi-step way, where the key factors inducing the occurrence and development of CRC are the activation of various oncogenes and the inactivation of tumor suppressor genes (26). In this study, we found that the low expression of SHP-2 induced by PHPS1 may regulate M2 polarization of TAMs and release of exosomes through PI3K/AKT pathway, thereby enhancing the migration and invasion ability of CRC cells (**Figure 9**).

SHP-2 is involved in the regulation of numerous tumor processes, but seems to play contradictory roles. Jiang et al. showed that hepatocellular carcinoma patients with low expression of SHP-2 had a lower 5-year survival rate and a poor prognosis (27). Helena et al. depicted that low expression of SHP-2 was associated with less favorable prostate cancer outcomes (28). In gastric cancer, low expression of SHP-2 was proved to be not associated with disease free survival in HCC patients (29). Here, our data demonstrated no correlation between SHP-2 expression and disease free survival and overall survival. The above interesting phenomenon can be explained from the heterogeneity of tumors, that is, the interaction between different TEMs, tumor cells, and tumor circulating cells affects patient prognosis. Additionally, SHP-2 as a phosphatase may recruit different substrates downstream to perform different functions under different conditions (8). On the other hand, there are data showing that the increased expression of SHP-2 was primarily detected in the hyperplastic epithelium and not in the lamina propria, indicating that SHP-2 may be involved early in the development of sporadic colorectal tumors (30).

Owing to the wide landscape of genomic alterations and limited therapeutic success in targeting tumor cells, recent studies have focused on better understanding and possibly targeting the microenvironment in which tumors develop. TAMs are among the most common tumor stromal cells in the TME and play significant roles in modulating the growth and metastasis of tumors. Functional polarization of TAMs to M1 or M2 represents a key mechanism that controls their functions, switching roles between tumor suppression and promotion (31). M2 polarization of macrophages is driven by multiple signaling pathways and cytokines such as IL-4, IL-13, and IL-10 (32). In our study, the characterization of macrophages produced *in vitro* showed that IL-4-conditioned macrophages exhibited a mixed M1/M2 phenotype with increased expression of Arginase-1, IL-10, TNF-α and IL-1β. Currently, most studies on the role of TAMs in tumor metastasis have focused on the M2 polarized phenotype. However, since monocyte/macrophage polarization is driven by environmental factors, TAMs may not be purely polarized M1 type or M2 type macrophages, but rather exhibited both pro- and anti-inflammatory properties (33). Indeed, Penny et al. also reported that TAM produced by pancreatic ductal adenocarcinoma expressed both M1 and M2 markers (34). In addition, this study also demonstrated that low expression of SHP-2 further stimulated IL-4-induced M2 macrophages to produce IL-10 and Arginase-1, which in turn further aggravated macrophage M2 polarization and ultimately promoted CRC tumor invasion and metastasis. Similarly, this way of regulating IL-4 expression was also observed in the study by Han et al. They stated that CENDE stimulated TAMs to produce IL-10 and Arginase-1, which then induced M2 polarization of TAMs to mediate the pro-tumor effect of CENDE (35). Collectively, these data demonstrate that M2-like TAM is an important mediator of low expression of SHP-2 to promote CRC progression.

PI3K was identified as an oncogenic gene to transform normal cells into cancer cells. It has been established that PI3K exerts its effects *via* activating the downstream protein AKT in a cascade manner (36). PI3K signaling is involved in a wide variety of cellular processes, including cell proliferation, survival, metabolism, and immunity (37). Interestingly, it has reported that PI3K-Akt pathway mediates polarization of monocytes into M2 macrophage through enhancing anti-inflammatory cytokine expression (38). Consistently, in our study, we found that low expression of SHP-2 activated PI3K/AKT pathway in M2 macrophages, as indicated by an increase in p-PI3K, p-AKT, Arginae-1 and IL-10. Notably, when PI3K/AKT pathway inhibitor LY294002 was added, the expression of the above proteins was significantly reduced, further confirming that low expression of 2 could promote M2 polarization through PI3K/AKT pathway and further affect CRC. This similar regulatory mechanism was also found in the study of Lian et al. (36). Their results revealed that epidermal growth factor which was secreted by colon cancer cells play contributory role in M2 polarization of macrophages through activation of PI3K/AKT pathway and secretion of cytokines.

Exosomes are extracellular vesicles derived from cells and have diameters of 30-200 nm and could modulate metastasis, angiogenesis, and drug resistance (39). The available data suggest that the polarization of M2 macrophages induced by tumor-derived exosomes can promote metastasis of CRC (15). Here, we found that low expression of SHP-2 promoted exosome release in IL-4-induced M2 macrophages by activating the PI3K/AKT pathway, and was inhibited after adding LY294002, suggesting that low expression of SHP-2 may also promote the release of exosomes in M2 macrophages through the PI3K/AKT pathway and thus affect CRC.

## Conclusion

Pharmacological inhibition the phosphatase activity of SHP-2 could serve as potential therapeutic targets of CRC. Further studies are now underway in order to define the precise mechanistic basis for how SHP-2 regulates PI3K, which should further our understanding of how SHP-2 couples this pathway to the regulation of cell survival. Besides, we will further analyze the role of SHP-2 in colorectal cancer by incorporating more clinical samples in further studies.

## Data availability statement

The original contributions presented in the study are included in the article/supplementary materials. Further inquiries can be directed to the corresponding author.

## Author contributions

ZZ conceived the study. ZHL, JX, BL and YL analyzed and interpreted the data. GW, BY, HM and ZLL collected and assembled the data. All authors read and approved the final manuscript.
